# Beyond the Glands: An in-Depth Perspective of Neurological Manifestations in Sjögren’s Syndrome

**DOI:** 10.4172/2161-1149.S4-010

**Published:** 2014-12-29

**Authors:** Alexandria Voigt, Sukesh Sukumaran, Cuong Q Nguyen

**Affiliations:** 1 Department of Infectious Diseases and Pathology, College of Veterinary Medicine, University of Florida, 2015 SW 16th Ave, Gainesville, Florida, USA; 2 Rheumatology Section, University of Arkansas for Medical Sciences, Arkansas Children's Hospital, Little Rock, Arkansas, USA; 3 Center for Orphan Autoimmune Disorders, University of Florida College of Dentistry, 1600 SW Archer Rd, Gainesville, Florida, USA

**Keywords:** Glands, Neuropathy, Sjögren’s syndrome

## Abstract

Primary Sjögren’s Syndrome (pSjS) is an autoimmune disease characterized by sicca (xerophthalmia or xerostomia) symptoms, anti-SS-A (Ro) or anti-SS-B (La) autoantibodies, and lymphocytic infiltrates in the exocrine glands. Disease incidence is estimated to be 0.1–3% of the general population with 0.4–3.1 million individuals in the US with women being nine times more likely to be afflicted with SjS than men. The frequency continues to rise accompanied with the multi-factorial etiology making it a challenging disease to manage and treat. Treatment of this disease remains problematic due to the lack of therapeutic treatments relying on replacement therapies such as artificial saliva and eye lubricants or immunosuppressive agents. To further complicate the management of the disease, there are number of multi-systemic manifestations specifically peripheral neuropathy associated with later stage of disease onset. Increasingly, there is mounting evidence that suggests the involvement of central nervous system. It remains to be determined the underlying cause and effect of the dysregulated immune response and the neuropathy associated with SjS. In this review, we provided an in-depth look at key neurological dysfunctions documented to occur in pSjS. Specifically, we discussed the prevalence, symptomology, and current treatments.

## Introduction

Sjögren's Syndrome (SjS) is an autoimmune disease characterized primarily by exocrine gland dysfunction, specifically of the salivary and lacrimal glands, resulting in dry mouth and dry eyes symptoms [1–4]. When it occurs in the absence of other autoimmune disorder, it is classified as primary SjS (pSjS). In secondary SjS (sSjS), the disease is associated with another autoimmune disorder, frequently Multiple Sclerosis (MS), Rheumatoid Arthritis (RA) or Systemic Lupus Erythematosus (SLE) [5–7]. Unlike most autoimmune connective tissue diseases, SjS shows the highest sexual dimorphism with women are affected 10-times more frequently than men, suggesting a role for sex hormones in disease susceptibility or progression [8–12]. One common feature of SjS in both humans and animal models is infiltration of mononuclear cells into the salivary and lacrimal glands, aggregating into clusters referred to as Lymphocytic Foci (LF) [13,14]. It can be systemic, affecting other organs including the gastrointestinal tract, skin, lungs, vasculature, kidneys, bladder and vagina. Involvement of the musculature can lead to fibromyalgia-like symptoms and chronic fatigue. Approximately 20% of patients develop various neuropathies, including sensory, peripheral, cranial and myelopathic neuropathies exhibited by cognitive impairments such as dementia, lack of concentration, memory loss and various psychiatric disorders [15–17]. There is evidence of a neurological aspect to the disease similar to the headaches present in SLE and brain lesions in MS [18,19]. Peripheral neuropathy in pSjS is broadly recognized and significant evidence is accumulating to include Central Nervous System (CNS) symptoms. Understanding this disease in relation to all of its symptoms is paramount to providing an accurate understanding of its etiology and development of an effective treatment. As such, summarized here is the neurological research, both peripheral and central.

## Peripheral nervous system

Similar to CNS involvement, the frequency of Peripheral Nervous System (PNS) symptoms vary based on the patient population and the objectives of the studies. Tajima et al. showed that PNS symptoms were reported to occur as frequently as 87% in patients exhibiting any neuropathy [20]. In patients with peripheral neuropathies, symptoms may include numbness, paresthesia and/or pain in the extremities. According to a study using large cohorts [21], the authors further classified peripheral neuropathy symptoms as sensory ataxia, painful sensory neuropathy without sensory ataxia, and radiculoneuropathy. Sensory ataxia occurred in 39% of patients, of those half had pain; 28% had trigeminal involvement. Normal nerve conduction was generally observed, but half of the patients exhibited Sensory Nerve Action Potentials (SNAPs) in the median and sural nerves. A sural nerve biopsy was performed and densities of both demyelinated and myelinated (large and small) fibers were decreased to 75%, 18%, and 56%, respectively. Axonal degeneration was present in 30% of the patient cohorts. Painful sensory neuropathy without sensory ataxia was observed in only 20% of the patients, 44% of which had trigeminal involvement. Sural and median nerve involvement was reduced to 17% and 11%, respectively without other nerve conduction issues apparent. Axonal sprouts were absent and degeneration was observed in approximately 19% of fibers; demyelinated and myelinated (large and small) fibers were decreased to 33%, 65% and 41%, respectively. Notably, the demylinated fibers density was significantly different between these two neuropathies. Only 4% of patients exhibited radiculoneuropathy. In these patients, demyelination appears to be present in the sural nerve and Somatosensory Evoked Potentials (SEPs) were prolonged.

Currently, there is no specific therapy or target to alleviate the symptoms of peripheral neuropathies for patients with SjS. Most of the treatments are systemic with broad immunosuppressive or antiinflammatory effect. Treatment with anti-inflammatory corticosteroid alone diminished symptoms in 18% of sensory ataxic neuropathies (all other responses were lower). Interestingly, Intravenous Immunoglobulin (IVIg) treated patients responded positively to the infusion; specifically response was detected in 23% of sensory ataxia patients, 67% of painful sensory neuropathy and 100% of radiculoneuropathy patients. However, the outcome was dependent on symptom presentation, which indicates that the underlying mechanism for various peripheral neuropathies might be different. Therefore it is crucial to precisely define the neuropathy process in these patients. A study by Gorson et al. examined six patients with polyneuropathy who had been treated with IVIg and were subsequently transitioned to Rituximab, on the basis that Rituximab had alleviated symptoms in immune-mediated neuropathies, but it proved ineffective as an alternative to IVIg treatment [22]. Contrarily, a recent study by Mekinian et al. has shown that Rituximab was effective in treating peripheral neuropathy if cryoglobulinaemia was present [23]. These results indicate an effective therapy might need to target specific aspect of neuropathies and not the overall disease. One particular target that needs to be considered is the immune-mediated etio-pathology.

Examination of autopsies of two pSjS patients with PNS involvement, one with ataxia and one with painful ataxia revealed that the dorsal root ganglia neurons were both affected by infiltration of CD8+ T cells [24]. Interestingly, McKeon et al. have shown that nicotinic ganglionic acetylcholine receptor autoantibodies were not present in pSjS patients [25]. However, other studies have demonstrated the association of neural autoantibodies and PNS neuropathies. For examples, Piccolo et al. identified a patient with anti-Ma2/Ta antibodies causing PNS involvement resembling primary lateral sclerosis [26]. And, Owada et al. reported a patient who presented peripheral neuropathy, but identified autoantibodies against spinal motor neurons and cerebellar Purkinje cells [27]. These case reports may indicate necessity in researching the relationship between peripheral neuropathy and anti-CNS autoantibodies. Two other markers of PNS symptoms were recently identified as aβ2GPI and p-ANCA in a study of 12 sensorimotor polyneuropathy patients [28].

## Trigeminal neuropathy

In cases of trigeminal sensory neuropathy, patients experience facial numbness, paresthesia, or (rarely) pain with either uni- or bi-lateral involvement [29]. In one study, 15 patients exhibited trigeminal neuropathy (9 unilateral and 6 bilateral), but nerve conduction of the limbs appeared normal. However, there was evidence that trigeminal neuropathy delays the blink reflex, which could exacerbate xerophthalmia symptoms [30]. Biopsy of one of the patient’s trigeminal nerves showed normal physiology, which the authors hypothesized that it might be an indication of vasculitis. However, considering the number of reports of neuronal autoantibodies and the lack of evidence of vasculitis in this patient, the more likely underlying cause is an inflammatory response. There are a few other studies examining the trigeminal nerve involvement in SjS [20,29], which support the findings in the aforementioned paper, but there are no studies that contain a larger pool of patients with specific trigeminal nerve involvement. Currently, there are no treatments available for this individual symptom, nor have any been suggested.

## Headaches

The prevalence of headaches in pSjS remains controversial. Sampling a large cohort of patients, Tjensvoll et al. determined that [31] 71 pSjS patients were shown to not have headaches more frequently than normal control patients-approximately 50% in both cohorts. However, other studies have concluded that there are significant differences in prevalence of autoimmune diseases (SLE, pSjS) and headaches compared to healthy controls. Tjensvoll et al. [18] and Harboe et al. [32] concluded that the frequency of headache in SjS and SLE were equivalent. One major drawback of these two studies is the high variability in the results, which may be attributed to the variation in patient populations or in the dependence on patient self-reporting. Another limitation is that dementia, memory deficits, or cognitive dysfuction may be present in pSjS patients and only one of the cited studies examined their patients’ memories, but did not include a normal group for comparison [32]. The fundamental deficiency on the majority of the studies reporting on headaches in pSjS is the precise description or criteria for headaches. Based on the International Classification of Headache Disorder, there is a spectrum as to what qualifies as a headache, namely migraine, migraine aura, Tension Type Headache (TTH) and pure TTH, and a variable severity of those categories. One study [18] found that 35% of patients have migraines and 56% have TTHs (at equivalent frequencies to SLE). Tjensvoll et al. showed that approximately 80% of pSjS patients (vs 64% healthy) had headaches with MIDAS (p=0.003) and HIT-6 scores (p=0.0009), indicating that patients experienced headaches more frequently and more severe. Interestingly, in a separate study, Tjensvoll et al. determined that pSjS patients had significantly more chronic TTHs than control patients (at a frequency of 8:1) with no significant increase in the overall frequency of non-TTH headaches [31]. While the criteria used were the same, the aspects they reported on were diverse. It appears that migraines are not associated with pSjS, but TTH or chronic TTH might correlate with pSjS and not with healthy control subjects.

Notably, in the majority of patients presenting with headache, there are no Magnetic Resonance Imaging (MRI) abnormalities, leading to speculation that it could be the result of vasculitis or autoantibody interaction. While vasculitis is a long-documented symptom of this disease, autoantibody binding to the neuronal antigens remains to be further examined. Previously, it was found that autoantibodies will bind selectively to the dorsal root ganglia, while foregoing any other binding to the central nervous system or peripheral nervous system components [33]. The evidence of selective neuronal autoantibodies present in pSjS, complexed with evidence of blood-brain barrier compromise by Cerebrospinal Fluid (CSF)/Serum Albumin Index [31] in a fraction of these patients, leads to the hypothesis that autoantibodies targeting neurons might the immunological trigger of peripheral or central neuropathies in SjS patients.

## Brain lesions

It has also been suggested that lesions may mimic Neuromyelitis Optica (NMO), but in a study of 12 patients with CNS involvement, only one was found to produce anti-aquaporin-4-antibody, which is indicative of NMO [34]. In those patients with TTH and lesions, the lesions appear to have a stronger signal hyperintensity and occur more specifically in the infratentorial region, the dorsal root ganglia, and subcortical sites [35] in relation to normal patients with TTH; the frequency of lesions correlated to the duration of the lesions. Additionally, the periventricular lesions correlated to the Platelet Serotonin Level (PSL), inversely; low PSL has previously been shown to be lowered in pSjS patients, but without any associated symptomology [36].

Those patients who had a headache and had an abnormal MRI scan generally showed white matter lesions. In a number of patients, this actually leads to misdiagnosis with MS, which displays characteristic lesions similar to pSjS [19].

In some patients, presentation of these lesions precedes diagnosis with pSjS [37–41]. In one reported case, these lesions actually disappeared without any treatment [39] and in others, treatment with corticosteroids resolved the symptom [37,41]. Modifications have also been suggested for MRI criteria, such as Barkhof’s criteria, and while this is sufficient to distinguish from CNS vasculitis, it is not a useful tool in the case of pSjS and MS [19]. Additionally, Single Photon Emission Tomography (SPECT) was used to assess regional Cerebral Blood Flow (rCBF) as an alternative to MRI when the results are inconclusive. In 18 of 32 patients with normal MR images, the SPECT revealed lesions, most frequently in the parietal lobe [42].

For patients reporting memory deficit, dementia or cognitive dysfunction, these white matter lesions are always present [32,43–48]. In one case of dementia, an Electroencephalogram (EEG) agreed with the SPECT findings above, identifying in the parietal lobes short runs of theta activities; symptoms improved with treatment of corticosteroids [49]. This same treatment for another patient worked and others in the study (treated with 5 to 10 mg daily of Donepezil) also saw improvement and ceased cognitive decline [45]. Treatment with hydroxychloroquine was also effective against memory and concentration deficits [48]; contrarily, L-dopa has not improved patient symptoms [47]. In this study, 12 patients (out of 20 pSjS patients) were found to have dementia- the majority of which had parietal lobe lesions by SPECT, but this statistic is skewed drastically from its occurrence in the average population, as the patients were chosen at a memory clinic. In another study, five out of 25 pSjS patients were found to have dementia, while a total of 15 out of the 25 had some cognitive dysfunction. Patients were administered the Batterie Courte d’évaluation cognitive destinée aux patients souffrant de Sclérose En Plaques (BCcogSEP) and demonstrated cognitive slowing, short- and long-term memory deficit, attention dysfunction and executive impairment [43].

To focus more specifically on the cognitive defects that have been attributed to pSjS, one group administered a variety of tests and found that patients performed significantly worse at simple reaction time task and executive function (by the Wisconsin Card Sorting Test (WCST) [44]. In another study, comparing pSjS to SLE, 50% of pSjS patients had normal cognitive function, and 50% had dysfunction ranging from mild to severe, respectively (24%, 21%, 6%) [32]. One other group tested a cohort of 120 pSjS patients and found that 44% showed cognitive dysfunction, including significant deficits in executive function (by the Trail Making Test A and B and the Tower of London (planning)), and verbal memory (by the Rey auditory Verbal Learning Test) [46].

## Myelopathy

Myelopathy, or lesions on the spinal cord, occurs less frequently than brain lesions. Myelopathy is diagnosed when patients present with a CNS symptom which may include muscle weakness, motor deficits, paresthesia, or paraplegia, etc [50]. These lesions are speculated to be the result of a Human T-Lymphocytic Virus 1 or 2 (HTLV-1 or HTLV-2) infection, which some hypothesize may act as an environmental trigger for pSjS [51,52]. However, in some cases myelopathy can present in pSjS patients independent of HTLV-1 or HTLV-2 infection [53]. Examination of CSF in patients infected with HTLV revealed elevated levels of autoantibodies including: anti-SSA, anti-SSB, and antibodies against CNS antigens, gangliosides and phospholipids. However, other studies have not examined the significant relationship between myelopathy and autoantibodies in the CSF. Tajima et al. examined 21 pSjS patients with CNS neuropathy in which one exhibited myelopathy, indicating that the incidence is low, even among pSjS patients presenting CNS involvement [20]. Unlike brain lesions, treatment of myelopathy with corticosteroids is ineffective [20]. Corticosteroids appeared to be effective in slowing the progression of myelopathy, however it failed to alleviate symptoms or cause the lesion to resolve. Current studies have shown some improvement with corticosteroids in conjunction with cyclophosphamide or chlorambucil [50], with antiviral medications [54], with an immunosuppressant such as azathioprine [53] in alleviating muscle weakness and paraplegia.

## Conclusion

Peripheral neuropathies in SjS have been documented since the late 1940s, however within the last three decades, the emergence of central nervous system involvement has been observed and further complicates the complexity of neuronal manifestation in SjS development. Currently, there is no standard categorization of the neurological symptoms of pSjS. To simplify the diagnosis and possible therapeutic regiments, one potential suggestion [21] is to divide neuropathies into seven different groups (sensory ataxic neuropathy, painful sensory neuropathy with sensory ataxia, multiple mononeuropathy, multiple cranial neuropathy, trigeminal neuropathy, autonomic neuropathy and radiculoneuropathy). Standardization of these categories and the criteria to fulfill them will provide a more complete picture of both the individual patient’s disease and as well as the disease as a whole. This will also allow for further research into specific therapies for these symptoms and standardization of treatments.

Presently, the standard treatment of neuropathy is corticosteroids, a systemic anti-inflammatory agent to suppress the broad autoimmune response. However, multiple studies offered alternatives, dependent on the neuropathological manifestations present. For peripheral neuropathies, IVIg is a more effective treatment than corticosteroids (except, perhaps in the case of sensory ataxia patients). Rituximab also proved a strong alternative to IVIg in certain patients (specifically those suffering from cryoglobulinaemia). Rituximab could be effective in treating the peripheral neuropathy but its efficacy seemed independent of the PNS symptomology. Corticosteroids are highly effective in treating brain lesions (which may spontaneously resolve), but for patients with memory deficits or dementia, treatment with Donepezil or hydroxychloroquine proved to have some beneficial efficacy. Corticosteroids are the standard treatment for myelopathy, but when used alone, the symptoms do not improve. Treatment should be combined with cyclophosphamide or chlorambucil, antiviral medication (in the case of HTLV-1 or -2 infection), or an immunosuppressant such as azathioprine to alleviate symptoms; resolution of the lesion has not yet been shown to occur. To date, there are no treatments for the pSjS-associated headache.

Neuropathies associated with SjS may be underreported as they can convalesce prior to sicca symptoms or without the patient realizing they have sicca symptoms, which in either case generally removes the necessity to perform further testing [55]. However, misdiagnosis is common due to these reasons [56–60] and may prevent an appropriate course of treatment. Recognizing the extent to which neuropathies are present in pSjS may help both neurologists and rheumatologists correctly diagnose and treat this aspect of the disease.
